# Directly injected lentiviral vector–based T cell vaccine protects mice against acute and chronic viral infection

**DOI:** 10.1172/jci.insight.161598

**Published:** 2022-09-22

**Authors:** Takuya Tada, Thomas D. Norton, Rebecca Leibowitz, Nathaniel R. Landau

**Affiliations:** 1 Department of Microbiology and; 2 Division of Infectious Diseases, Department of Medicine, NYU Grossman School of Medicine, New York, New York, USA.

**Keywords:** Immunology, Vaccines, Dendritic cells, T cells

## Abstract

Lentiviral vector–based dendritic cell vaccines induce protective T cell responses against viral infection and cancer in animal models. In this study, we tested whether preventative and therapeutic vaccination could be achieved by direct injection of antigen-expressing lentiviral vector, obviating the need for ex vivo transduction of dendritic cells. Injected lentiviral vector preferentially transduced splenic dendritic cells and resulted in long-term expression. Injection of a lentiviral vector encoding an MHC class I–restricted T cell epitope of lymphocytic choriomeningitis virus (LCMV) and CD40 ligand induced an antigen-specific cytolytic CD8^+^ T lymphocyte response that protected the mice from infection. The injection of chronically infected mice with a lentiviral vector encoding LCMV MHC class I and II T cell epitopes and a soluble programmed cell death 1 microbody rapidly cleared the virus. Vaccination by direct injection of lentiviral vector was more effective in sterile alpha motif and HD-domain containing protein 1–knockout (SAMHD1-knockout) mice, suggesting that lentiviral vectors containing Vpx, a lentiviral protein that increases the efficiency of dendritic cell transduction by inducing the degradation of SAMHD1, would be an effective strategy for the treatment of chronic disease in humans.

## Introduction

Several features of lentiviral vectors have made them advantageous for the expression of therapeutic proteins in vivo. The vectors integrate preferentially into transcriptionally active sites in chromosomal DNA, resulting in the long-term expression of the encoded protein in the target cell and daughter cells (1, 2); they do not encode additional viral proteins; and they are generally not subject to preexisting immunity as occurs for other viral vectors (3). In addition, lentiviral vectors transduce nondividing cells (1, 4–6) and have a broad tissue tropism when pseudotyped by the vesicular stomatitis virus G (VSV-G) glycoprotein (7, 8).

These properties have made lentiviral vectors advantageous for use in dendritic cell (DC) vaccines. DC vaccines have been developed for cancer and infectious diseases. The approach takes advantage of the central role of DCs as antigen-presenting cells (APCs) in orchestrating primary immune response (9–16). In this approach, a patient’s monocytes are obtained by leukapheresis and then differentiated ex vivo to immature DCs. The DCs are then pulsed with synthetic peptide or cell lysate and matured with cytokine or TLR agonists. Alternatively, the DCs are transduced with a viral vector that expresses the antigen. Endogenous synthesis of antigen results in efficient proteolytic peptide processing and presentation of peptide antigen on MHC class I proteins. Vectors that have been used to express antigen in DCs, in addition to lentiviral vectors, include (17) adenovirus (18–21), adeno-associated virus (22–24), Sendai virus (25, 26), vaccinia virus (27, 28), and herpes simplex virus (29, 30). Lentiviral vector–based DC vaccines have proved successful therapeutically and prophylactically in animal tumor models and models of virus infection (31–34). The transduction of DCs with lentiviral vectors encoding peptide antigen has been found to induce potent antiviral CD8^+^ T cell responses (35).

An obstacle to the use of lentiviral vectors in DC vaccines lies in their low efficiency of myeloid cell transduction. Myeloid cells express sterile alpha motif and HD-domain containing protein 1 (SAMHD1), a host restriction factor that interferes with the lentiviral reverse transcription by depleting the intracellular pool of deoxynucleotide triphosphates, thereby decreasing the efficiency with which they are transduced by lentiviral vectors (36–39). Lentiviral transduction of DCs can be greatly enhanced by the incorporation of the lentiviral accessory protein Vpx into the virions. Virion-packaged Vpx is released from the virus upon virus entry, inducing the proteasomal degradation of SAMHD1, thereby relieving the block to reverse transcription (40, 41). HIV-based lentiviral vectors engineered to package simian immunodeficiency virus Vpx transduce DCs with a nearly 2-log increase in titer, increasing their usefulness in DC vaccines (35, 42).

We previously reported on a lentiviral vector–based DC therapeutic vaccine for HIV-1 infection using a vector that coexpressed the CD8^+^ T cell epitope together with CD40 ligand (CD40L) (35). CD40L served to mature and activate the DCs, causing increased expression of CD83 and CD86 (42) and the secretion of high levels of Th1 cytokines IL-12p70, TNF-α, and IL-6. Immunization of bone marrow liver fetal thymus–humanized mice with DCs that had been transduced with Vpx-containing virions induced a strong CD8^+^ T cell response and suppressed HIV-1 virus loads in mice (35). In addition, we reported on a lentiviral vector–based DC vaccine against lymphocytic choriomeningitis virus (LCMV) (43) based on a lentiviral vector expressing the LCMV CD8^+^ T cell epitope GP33 and a soluble programmed cell death 1 (PD-1) “microbody.” The microbody acted as a checkpoint inhibitor (44) by binding to the programmed cell death ligand 1 (PD-L1) on T cells, preventing its interaction with PD-L1 (44). Immunization with vector-transduced DCs induced a strong CD8^+^ T cell response that prevented lethality in acutely infected mice and was therapeutic in chronically infected mice (43).

In this study, we tested whether mice could be vaccinated by direct injection with a lentiviral vector, an approach that would obviate the need for ex vivo transduction and reinfusion. We found that direct injection of a lentiviral vector expressing LCMV CD8^+^ T cell epitopes, CD40L, and a PD-1 microbody checkpoint induced a strong cytotoxic T lymphocyte response that protected mice from lethal LCMV infection. Injection of the vector into chronically infected mice resulted in rapid clearance of the virus. The directly injected vector preferentially transduced splenic DCs and some B cells. The lentiviral vector vaccination was more effective in SAMHD1-knockout (KO) mice, suggesting the advantage of Vpx-containing lentiviral vectors for use in humans.

## Results

### Long-lived expression by direct injection of lentiviral vector.

**Figure 1:**
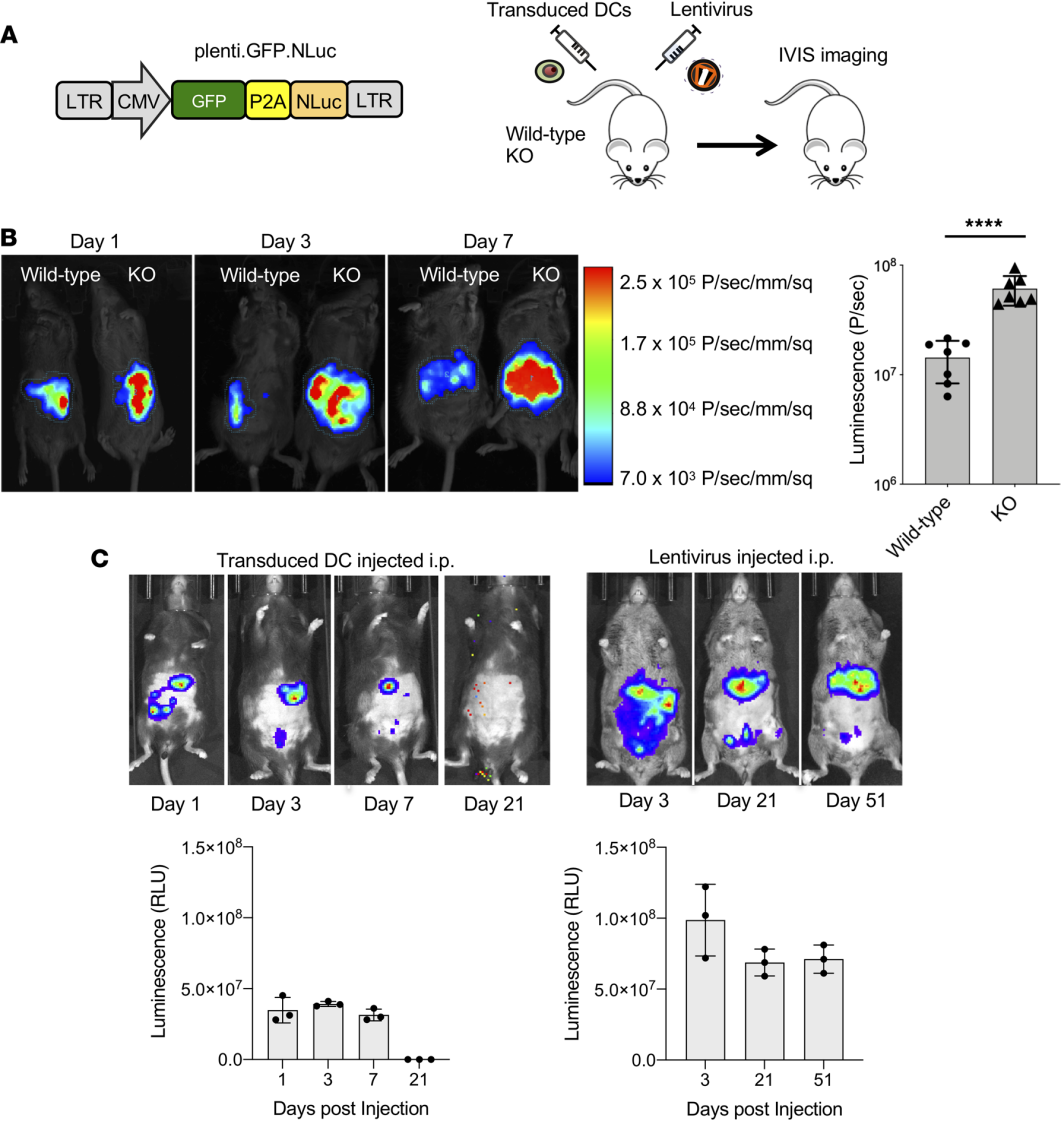
Direct lentiviral vector injection extends the in vivo half-life of transduced cells. (**A**) Diagram of the GFP/luciferase lentiviral vector GFP-NLuc (left). Wild-type and SAMHD1-knockout (KO) mice were injected i.p. with 1 × 10^7^ infectious units (IU) of VSV-G–pseudotyped GFP-NLuc virus and imaged over time on an in vivo imaging systems (IVIS) imager as diagrammed (right). (**B**) Wild-type and SAMHD1-KO mice were injected with GFP/luciferase lentiviral vector and imaged at 1, 3, and 7 days postinjection (left). The image is pseudocolored according to luciferase signal intensity as shown on the bar at the right with colors corresponding to photons/s/mm^2^. Luciferase activity on day 7 (*n* = 6) was quantified by IVIS (right). Statistical significance was determined by 2-tailed, unpaired *t* test. Confidence intervals are shown as the mean ± SD. (****P *≤* 0.0001.) (**C**) SAMHD1-KO mice (*n* = 3) were injected with GFP-NLuc vector–transduced BMDCs (1 × 10^6^ cells) or injected i.p. with GFP-NLuc virus (2 × 10^7^ IU) and imaged on the indicated days. Luciferase activity was quantified as shown below.

To determine the feasibility of using lentiviral vectors for vaccination by direct injection, we constructed the lentiviral reporter vector plenti.GFP-NLuc that coexpresses a GFP:nanoluciferase fusion protein separated by P2A self-processing peptide motif (Figure 1A) and used the vector to determine the durability of expression and to identify the cell types transduced by direct injection. In addition, we tested how these parameters would be affected by the absence of SAMHD1. In tissue culture, SAMHD1-KO mouse myeloid cells are more efficiently transduced by lentiviral vectors; whether this increase occurs in vivo in mice is not clear (35). It is not possible to answer this question in mice using lentiviral vectors because the interaction of Vpx with SAMHD1 is species specific, and as a result, Vpx does not induce the degradation of murine SAMHD1. We therefore modeled the effect of Vpx in humans by using SAMHD1-KO mice. GFP-NLuc virus was injected intraperitoneally (i.p.) into wild-type and SAMHD1-KO mice, and luciferase expression was visualized on days 1, 3, and 7 by IVIS spectrum live imaging. The results showed that luciferase expression was 4.2-fold higher in the SAMHD1-KO as compared with wild-type mice and that the level of expression increased over the time course (Figure 1B). The results demonstrate that lentivirus transduction in vivo is limited by SAMHD1.

To compare the half-life of expression by injected ex vivo–transduced DCs with that resulting from directly injected lentiviral vector, SAMHD1-KO bone marrow–derived DCs (BMDCs) were transduced with GFP-NLuc vector and injected i.p. into SAMHD1-KO mice, and, in parallel, the vector was injected i.p. Imaging of the mice over the next 51 days showed the injected BMDCs localized to the upper left quadrant of the abdomen, a region corresponding to the spleen. The cells were relatively short-lived, becoming nearly undetectable by day 21 (Figure 1C). Direct injection of the vector resulted in much longer term expression, also localized to the spleen, that was maintained at high levels through day 51 (Figure 1C). An analysis of luciferase activity in different organs showed the highest level of expression in the spleen and liver and low-level expression in lymph node, kidney, brain, intestine, and lung (Supplemental Figure 1; supplemental material available online with this article; https://doi.org/10.1172/jci.insight.161598DS1). The tissue distribution was similar in mice injected intravenously (i.v). The results demonstrated the much longer term expression that resulted from injection of the lentiviral vector as compared with injection of DCs.

### Transduction of APCs by direct injection.

**Figure 2:**
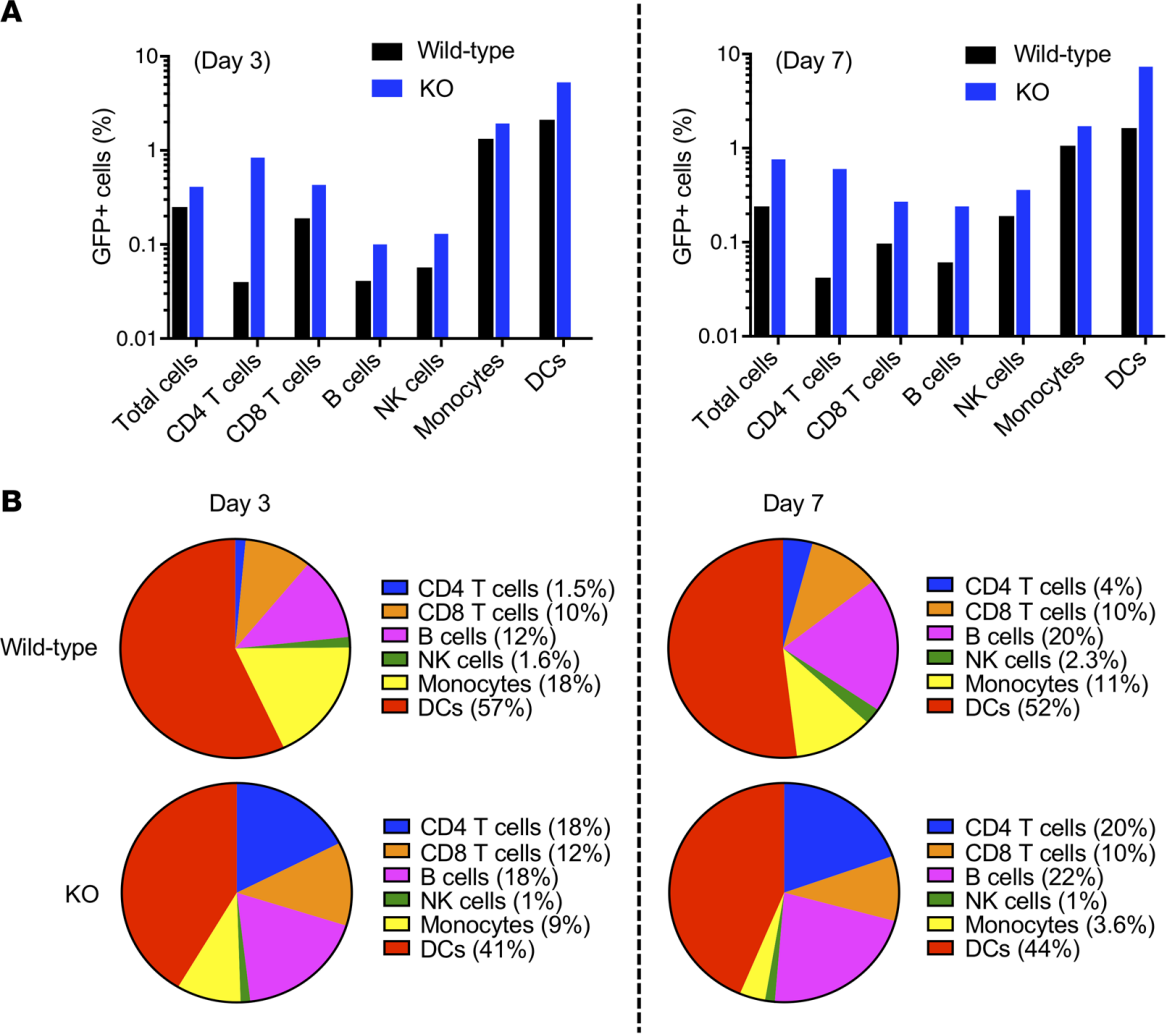
APCs, including B cells, monocytes, and DCs, were transduced. (**A**) Wild-type and SAMHD1-KO mice injected with 3 × 10^7^ IU of GFP-expressing lentivirus. After 3 (left) or 7 days (right), the mice were sacrificed, and splenocytes were stained with antibodies against CD3, CD4, CD8, CD19, CD49b, CD11c, CD115, and MHCII and analyzed by flow cytometry. The proportion of GFP^+^CD4^+^ T cells (CD3^+^CD4^+^), CD8^+^ T cells (CD3^+^CD8^+^), B cells (CD3^–^CD19^+^), NK cells (CD3^–^CD49b^+^), monocytes (CD115^–^CD11b^+^), and DCs (CD11c^+^MHCII^+^) was quantified as shown in the histograms. (**B**) The fraction of GFP^+^ cells of each of the cell types for the wild-type and SAMHD1-KO mice on day 3 (left) and day 7 (right) is shown as a pie chart with the percentage of each cell type in parentheses.

To determine the cell types transduced by direct injection, wild-type and SAMHD1-KO mice were injected i.p. with GFP-NLuc. The mice were sacrificed 3 and 7 days later, and the splenocytes were analyzed by flow cytometry to determine the cell types of the GFP^+^ cells using a panel of antibodies to classify the cells as CD4^+^ T cells (CD3^+^CD4^+^), CD8^+^ T cells (CD3^+^CD8^+^), B cells (CD3^–^CD19^+^), NK cells (CD3^–^CD49b^+^), monocytes (CD115^–^CD11b^+^), and DCs (CD11c^+^MHCII^+^) (Figure 2A). On day 3 postinjection, approximately 0.25% of splenocytes from wild-type mice and 0.4% from SAMHD1-KO mice were GFP^+^. On day 7 postinjection, approximately 0.25% and approximately 0.8% of splenocytes from wild-type and KO mice, respectively, were GFP^+^. All the cell types of the SAMHD1-KO mice were transduced with higher efficiency than those of wild-type mice (Figure 2B). The cell type with the highest representation was the DCs, both for KO and wild-type mice. B cells and monocytes, which also serve as APCs, were also transduced. The cell type for which the transduction frequency increased the most because of the SAMHD1-KO mice was the CD4^+^ T cells, which were 12-fold higher in the KO mice on day 3 and 5-fold increased at day 7 as compared with wild-type. This finding is consistent with the reported role of SAMHD1 in restricting HIV infection of resting T cells (45). The distribution of cell types transduced was similar on day 7 and similar in mice injected i.v. though with fewer transduced cells (data not shown).

### Directed injection of lentiviral vaccine protects against acute infection.

**Figure 3:**
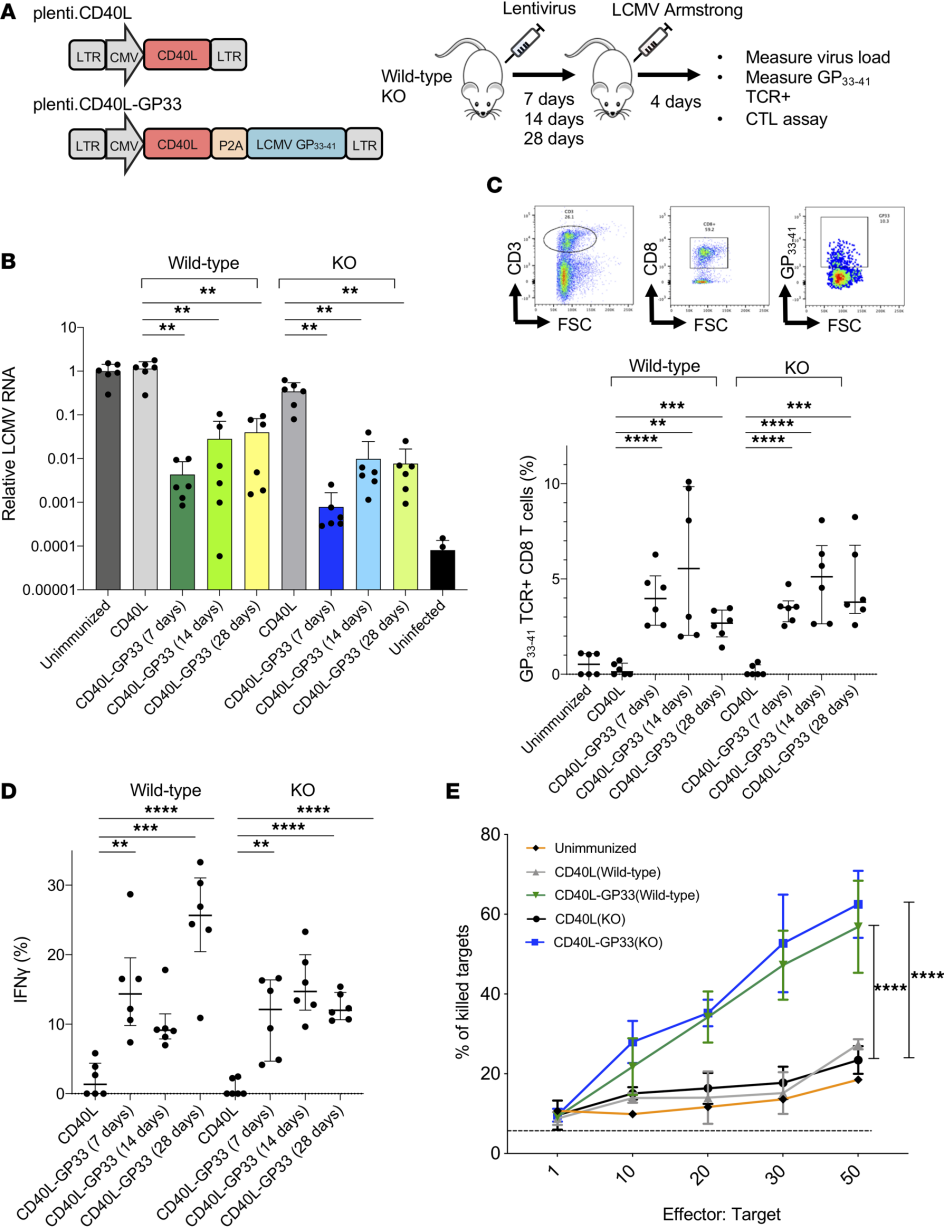
Direct injection of lentiviral vaccine protects against LCMV infection. (**A**) The structures of the lentiviral vaccine vector CD40L-GP33 expressing CD40L and LCMV MHCI epitope GP_33-41_ (left) are shown. Wild-type and SAMHD1-KO mice (*n* = 6) were injected i.v. or i.p. with 3 × 10^6^ IU of the viruses, and after 1, 2, or 4 weeks, the mice were challenged with LCMV Armstrong virus (right). Virus load, TCR^+^CD8^+^ T cells, and cytolytic activity were measured 4 days postinfection. (**B**) LCMV RNA copy numbers in the spleen of the immunized mice were quantified by quantitative reverse transcription PCR (qRT-PCR) (*n* = 6) 4 days postinfection. (**C**) Splenocytes from mice challenged 2 weeks postimmunization were stained with GP33 tetramer and antibodies against CD3 and CD8. CD3^+^CD8^+^ cells were gated, and the number of tetramer^+^ cells was determined. The gating strategy is shown (above) and the percentage of tetramer^+^CD8^+^ T cells was quantified. (**D**) The proportion of splenic GP33^+^TCR^+^CD8^+^ T cells expressing IFN-γ was quantified by flow cytometry. (**E**) Cytolytic activity of splenocytes from the immunized mice (*n* = 6) was measured in vitro. LB27.4 target cells were stained with CFSE and pulsed with GP33 peptide and then incubated for 24 hours with different numbers of effector splenocytes. The cells were then stained with viability dye eFluor 450 and the dead cells quantified by flow cytometry. Statistical significance was determined by Kruskal-Wallis test with post hoc Dunn’s test. Confidence intervals are shown as the mean ± SD. (**P *≤* 0.01, ***P *≤* 0.001, ****P *≤* 0.0001.)

The infection of ex vivo–transduced DCs protects mice against infection with LCMV (43). To determine whether mice could be vaccinated by directed injection, wild-type and SAMHD1-KO mice were injected with CD40L-GP33 (0.3 × 10^6^ to 10 × 10^6^ IU), a lentiviral vector that encodes the LCMV MHC class I–restricted GP_33-41_ epitope (GP33) and murine CD40L, or with control vector plenti.CD40L that encodes only murine CD40L. After 7, 14, and 28 days, the mice were challenged with LCMV Armstrong. Splenic viral loads were quantified 4 days postchallenge (Figure 3A). The results showed that immunization with plenti.CD40-GP33 7, 14, or 28 days prior to LCMV challenge suppressed the virus load 23-fold, 10-fold, and 104-fold, respectively, in wild-type mice and 1360-, 72-, and 55-fold, respectively, in SAMHD1-KO mice (Figure 3B). The nearly 60-fold difference in viral load reduction between SAMHD1 KO and wild-type mice immunized 7 days before LCMV challenge suggests that the immune response was induced more rapidly in the SAMHD1-KO mice. Immunization 14 and 28 days prior to LCMV challenge resulted in less virus load suppression than immunization 7 days prior to challenge but was 100-fold lower than controls. Injection of the control plenti.CD40L vector caused a small increase in virus loads that may have been caused by the induction of replication-enhancing cytokines by CD40L. Protection from infection required inoculation with as little as 0.3 × 10^6^ IU (Supplemental Figure 2A). Vaccination by direct injection was somewhat less effective than vaccination with transduced DCs, which resulted in a slightly greater suppression of virus loads (Supplemental Figure 2A). A vector that expressed an inactive CD40L carrying a T146N mutation (mutCD40L-GP33) (46) was less active in stimulating an antiviral response, demonstrating the role of the accessory protein in stimulating the T cell response to vaccination (Supplemental Figure 2A). Vaccination by i.p. injection rather than i.v. was also effective but resulted in a less pronounced suppression of virus loads (>1 log) (Supplemental Figure 2B).

To determine the number of antigen-specific CD8^+^ T cells induced by vaccination, mice were immunized by injection of the GP33/CD40L-expressing lentiviral vector and challenged with LCMV 7, 14, and 28 days postimmunization. Four days postchallenge, the number of antigen-specific CD8^+^ T cells was determined by flow cytometry analysis of splenocytes stained for CD3 and CD8 and with a GP33/MHC class I tetramer (Figure 3C). The analysis showed that immunization with the CD40L-GP33 vector resulted in increased numbers of antigen-specific TCR^+^CD8^+^ T cells as compared with unimmunized or CD40L control vector–immunized mice in which antigen-specific T cells were not detectable. The number of tetramer^+^ cells decreased over the time course in the wild-type mice but remained constant in the SAMHD1-KO mice. The numbers of IFN-γ^+^CD8^+^ T cells increased to a similar extent in the wild-type and SAMHD1-KO mice and remained constant over the 28-day time course (Figure 3D). Measurement of IFN-γ levels in the serum showed similar increases (Supplemental Figure 3). Monocyte chemoattractant protein-1 (MCP-1) and TNF-α levels decreased (Supplemental Figure 3). Measurement of the T cell cytolytic activity showed that the T cells of vaccinated wild-type and SAMHD1-KO mice had potent cytolytic activity (Figure 3E).

### Adoptively transferred CD8^+^ T cells and DCs suppress LCMV load.

**Figure 4:**
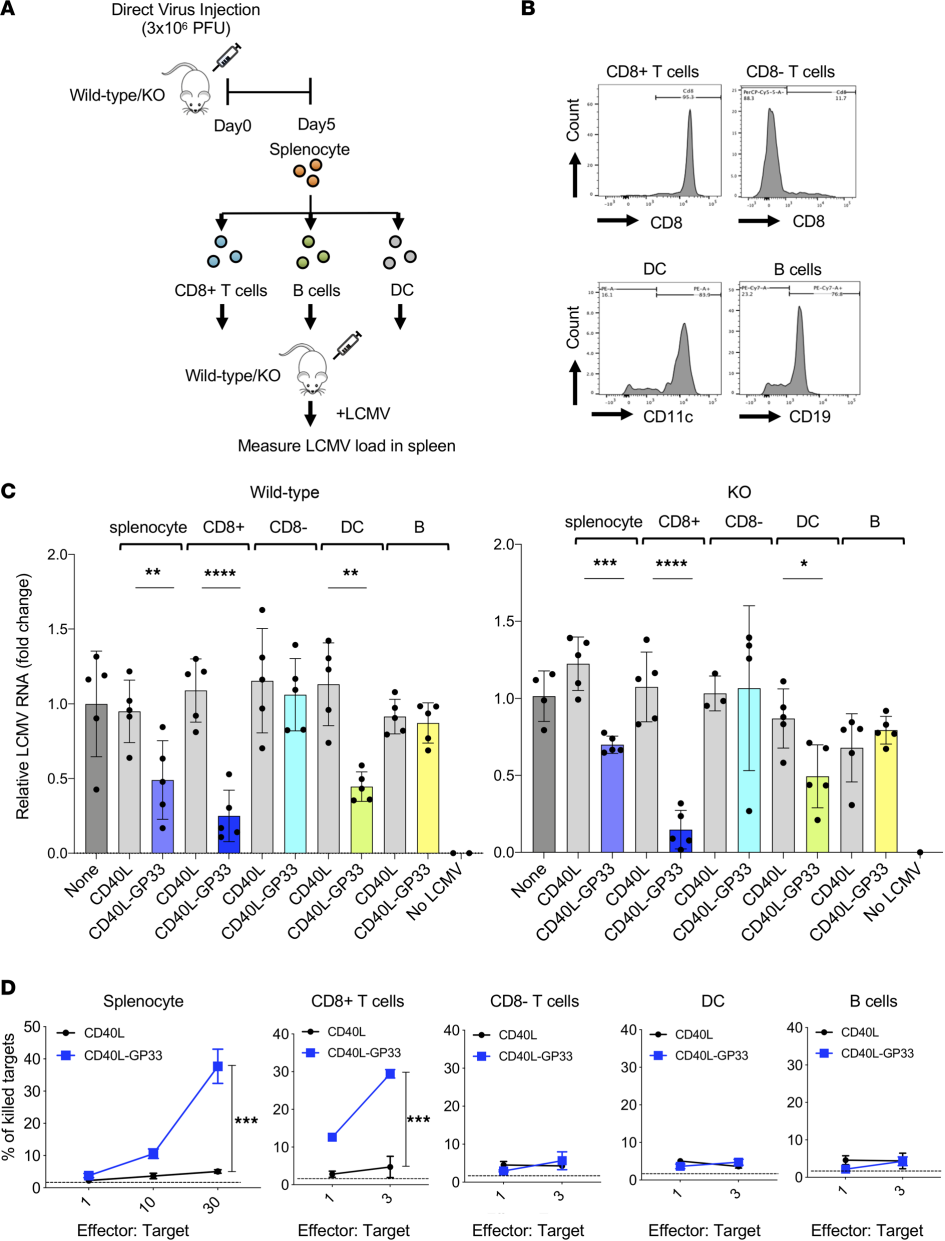
Adoptively transferred CD8^+^ T cells and DCs suppress virus loads. (**A**) As diagrammed, wild-type or SAMHD1-KO mice were immunized by i.p. injection of CD40L-GP33 or control CD40L virus. Five days postimmunization, the mice were sacrificed, and splenic CD8^+/–^ T cells, DCs, and B cells were isolated on magnetic beads. The cell populations were injected i.v (*n* = 5) into wild-type or SAMHD1-KO mice, and 3 days later, the mice were challenged with LCMV Armstrong. (**B**) Isolated CD8^+/–^ T cells, DCs, and B cell populations from SAMHD1-KO mice were stained with antibodies against CD8, CD11c, and CD19 analyzed by flow cytometry. (**C**) Wild-type (left) and SAMHD1-KO (right) mice (*n* = 5) were adoptively transferred with total splenocytes, CD8^+^ T cells, CD8^–^ T cells, DCs, and B cells from mice immunized with CD40L or CD40L-GP33 virus. Controls included mice that were not adoptively transferred and mice that were not infected. LCMV RNA copy numbers were quantified day 4 postinfection by qRT-PCR. (**D**) Five days after adoptive transfer, CD8^+/–^ T cells, DCs, and B cells were isolated from the SAMHD1-KO spleen, and cytolytic activity was measured in vitro. Effector cells were incubated with CFSE-stained, GP33-coated LB27.4 target cells for 24 hours. The number of dead target cells was quantified by flow cytometry. Statistical significance was determined by 2-tailed, unpaired *t* test. Confidence intervals are shown as the mean ± SD. (*P *≤* 0.05, **P *≤* 0.01, ***P *≤* 0.001, ****P *≤* 0.0001.)

To determine which cell types mediated the antiviral response, wild-type and SAMHD1-KO mice were immunized with CD40L-GP33 or control plenti.CD40L virus, and after 1 week, CD8^+^ T cells, CD8^–^ T cells, DCs, and B cells were isolated from the spleen (Figure 4A). Analysis of the purified populations by flow cytometry showed that the populations were highly enriched for their respective cell types (Figure 4B). The unpurified splenocytes and the individual purified populations from wild-type and SAMHD1-KO mice were injected into mice, and 3 days postinjection, the mice were challenged with LCMV Armstrong. Quantification of virus loads 4 days postchallenge showed that unpurified splenocytes from mice injected with the control CD40L virus caused a small 1%–10% increase in virus loads while splenocytes from CD40L-GP33 virus caused a minor 25% to 50% decrease in virus loads both in wild-type and in SAMHD1-KO mice. CD8^+^ T cells caused a more pronounced 10-fold decrease. CD8^–^ T cells and B cells had no effect while DCs caused a small but significant decrease in virus loads in both the wild-type and SAMHD1-KO mice (Figure 4C). Analysis of cytolytic activity of the purified populations showed killing of target cells by the unpurified splenocytes and by the CD8^+^ T cells but not by the CD8^–^ T cells, DCs, or B cells (Figure 4D). These results suggest that the antiviral response was largely due to CD8^+^ T cell cytolytic activity and that DCs and B cells were able to prime the antiviral response in recipient mice.

### Lentiviral therapeutic immunization for chronic infection.

**Figure 5:**
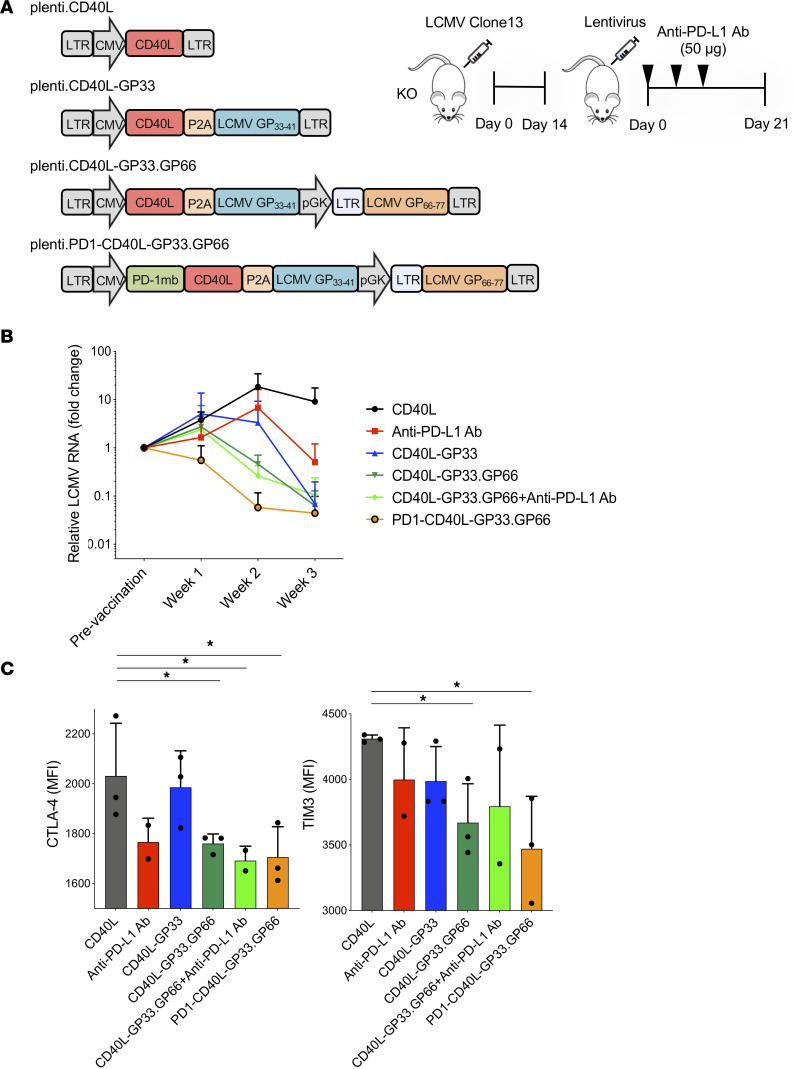
Direct lentivirus immunization cures chronic LCMV infection. (**A**) The structures of the lentiviral vaccine vector CD40L; CD40L-GP33; CD40L-GP33.GP66 expressing CD40L, LCMV MHC class I epitope GP_33-41_, and LCMV MHC class II epitope GP_66-77_; PD-1-CD40L-GP33.GP66 expressing PD-1 microbody, CD40L, LCMV MHC class I epitope GP_33-41_, and LCMV MHC class II epitope GP_66-77_ were shown. SAMHD1-KO mice were infected with 3 × 10^6^ PFU of LCMV clone 13. Two weeks after LCMV infection, mice were immunized with 3 × 10^6^ IU of lentivirus (*n* = 3). A total of 50 μg of PD-L1 antibody was injected into mice every 3 days. (**B**) Virus loads in the serum were measured weekly. (**C**) Analysis of CTLA-4 and T cell immunoglobulin mucin receptor 3 (TIM3) levels on the antigen-specific CD8^+^ T cells. Statistical significance was determined by Kruskal-Wallis test with post hoc Dunn’s test. Confidence intervals are shown as the mean ± SD. (*P *≤* 0.05.)

We previously reported that DCs transduced ex vivo and reinjected could suppress virus loads in SAMHD1-KO mice chronically infected with LCMV clone 13 (43) and that the suppression was more effective in combination with a checkpoint inhibitor antibody. A vector encoding GP33 in combination with the MHC class II–restricted epitope GP_66-77_ (plent.CD40L-GP33.GP66) suppressed virus loads better, and the inclusion of a soluble form of PD-1 termed a PD-1 microbody in the vector plenti.PD1-CD40L-GP33.GP66 was also more effective (43). To determine whether a similar effect of chronic infection could be achieved by direct injection of the lentiviral vector, we established mice chronically infected with LCMV clone 13 and then vaccinated them with control plenti.CD40L, plenti.CD40L-GP33, plenti.CD40L-GP33.GP66, CD40L-GP33.GP66, or plenti.PD-1-CD40L-GP33.GP66. In one group the mice were vaccinated with plenti.CD40L-GP33.GP66 and subsequently treated with anti–PD-L1 antibody. As a control, mice were injected with the antibody without vaccination. Serum virus loads were measured weekly (Figure 5A). The results showed that in mice immunized with control CD40L vector, virus loads remained high over the 3-week time course. The injection of anti–PD-L1 antibody caused a 10-fold decrease in virus loads. The CD40L-GP33 vector caused a similar 10-fold decrease. The dual CD40L-GP33.GP66 vector caused a significantly greater decrease in virus load. The addition of the checkpoint inhibitor antibody had little effect. The most pronounced decline in virus loads was caused by the plenti.PD-1-CD40L-GP33.GP66 vector that encoded both epitopes together with the PD-1 microbody (Figure 5B). Analysis of cell surface exhaustion marker cytotoxic T-lymphocyte-associated protein 4 (CTLA-4) in the LCMV-specific CD8^+^ T cells showed anti–PD-L1 antibody decreased expression of the marker. The single epitope vector had little effect, but the dual-epitope vectors significantly decreased expression (Figure 5C). Similar results were found for the exhaustion marker TIM3, where the most pronounced decrease in expression was caused by the vector that expressed both epitopes and the PD-1 microbody (Figure 5C). Although CTLA-4 is induced in T cells by activation, T cells activated in the presence of a checkpoint blockade maintain low CTLA-4 levels (47). The results demonstrated that a vector encoding class I– and class II–restricted epitopes along with a checkpoint inhibitor was an effective strategy to prevent checkpoint activation and increase the immune response against chronic infection.

## Discussion

The injection of mice with a lentiviral vector–based vaccine encoding CD40L and a CD8^+^ T cell epitope protected mice against acute LCMV infection and rapidly cleared virus from chronically infected mice. Lentiviral vector direct injection primarily targeted DCs in the spleen and resulted in long-term expression of the encoded protein. The results demonstrated that an effective T cell response could be generated by direct injection of the vector without the need for ex vivo transduction of DCs. Injection of as little as 0.3 × 10^6^ IU of the vector i.p. or i.v. protected the mice from LCMV infection and prevented the induction of proinflammatory cytokines TNF-α, MCP-1, and IL-6. The vaccine-induced CD8^+^ T cells expressed IFN and had high cytolytic activity on antigen-expressing target cells. The findings are consistent with those of Esslinger et al., who previously reported that footpad injection of a lentiviral vector transduced cells in the draining lymph node and spleen and that DCs were frequently targeted (48). While T cell priming is thought to mainly occur in secondary lymphoid organs, we did not detect transduced cells in the lymph node, suggesting that the spleen is also a site in which T cells can be primed.

SAMHD1-KO mice were transduced more efficiently by the injected lentiviral vector than wild-type mice, and a more protective T cell response was induced. Moreover, in SAMHD1-KO mice, the numbers of antigen-specific CD8^+^ T cells remained constant over time while they deceased in wild-type mice. Several leukocyte cell types were transduced to a higher extent in the SAMHD1-KO mice, suggesting that SAMHD1 plays a role in restriction of cell types in addition to myeloid cells. The cell type for which transduction was most increased by the absence of SAMHD1 was the CD4^+^ T cells. Lentiviral infection of human resting T cells in culture was shown to be restricted by SAMHD1, consistent with this finding (45). While it is not possible in mice to directly test whether packaging of Vpx in the vector would be advantageous, the increased transduction efficiency of the SAMHD1-KO mice suggests that in clinical use, Vpx-containing vectors would result in the transduction of increased numbers of DCs. Adoptive transfer demonstrated that the vaccine-induced CD8^+^ T cells were sufficient to suppress virus replication. DCs from vaccinated mice were able to induce a protective immune response, demonstrating the potency of the DCs, only 2% of which had been transduced.

The injected vaccine rapidly suppressed virus loads in chronically infected mice. The effect was more pronounced by the inclusion of an MHC class II–restricted epitope in the vaccine vector together with a PD-1 microbody. The results suggest the value of combining class I and class II epitopes and the benefit of encoding the checkpoint inhibitor in the vaccine vector itself. Coinjection of anti–PD-L1 antibody did not have this effect, suggesting that the presence of the checkpoint inhibitor at the site of T cell activation results in more active T cells. The PD-1 microbody served to saturate PD-1 on the DC surface, thereby preventing it from signaling to PD-L1 on T cells and decreasing checkpoint activation (49, 50). The increased functionality of the CD8^+^ T cells was reflected by their decreased levels of exhaustion markers CTLA-4 and TIM3. The PD-1 microbody was previously shown to suppress HIV-1 replication in a humanized mouse model using DCs that had been transduced ex vivo and reinjected (35).

A concern for the clinical use of lentiviral vector encoding CD40L and a PD-1 microbody is that of an inflammatory response caused either by the encoded proteins or as a result of vector integration. CD40L induces DCs to express proinflammatory cytokines, such as TNF-α and IL-6 (51), and checkpoint inhibitors can also induce inflammation. However, injection of the vector did not cause an inflammatory response or result in any apparent deleterious effects on the health of the mice. The mice did not show any changes in body weight, hair coat, ocular discharge, or breathing; there were no discernable effects on leukocyte subsets in blood, lymph node, and spleen or evidence of cellular activation or increases in levels of proinflammatory cytokines (not shown).

The findings shown here suggest that direct injection of a lentiviral vector–based vaccine can induce a potent T cell response. Direct injection resulted in much longer term antigen expression than ex vivo–transduced DCs, an effect that could be advantageous to continually stimulate T cell responses in chronic disease where T cell exhaustion diminishes the effectiveness of T cells. The approach could be useful both in the treatment of HIV-1 infection as well as in cancer, both of which are diseases in which T cell exhaustion plays an important role in disease severity. In cancer, immunotherapy checkpoint inhibitor antibodies reverse T cell exhaustion but are associated with autoimmune inflammation. The continuous low-level expression of a checkpoint inhibitor by APCs could circumvent this issue. In conclusion, direct injection of a lentiviral vector–based vaccine could be useful as a therapeutic vaccine for individuals on long-term treatment for chronic infection with HIV-1 or as a cancer vaccine in combination with a checkpoint inhibitor.

## Methods

### Cells.

HEK293T (ATCC) cells were cultured in DMEM/10% FBS at 37°C in 5% CO_2_. LB27.4 (from Marcel van den Brink, Memorial Sloan Kettering Cancer Center, New York, New York, USA) were cultured in RPMI 1640 (Corning) with 10% FBS, 2 mM l-glutamine, and 50 μM 2-mercaptoethanol. Murine BMDCs were prepared by extracting bone marrow cells from the hind legs of 6- to 12-week-old mice. The cells (5 × 10^6^) were differentiated in a 15 cm culture dish in RPMI 1640, 10% FBS, 1 mM sodium pyruvate, 2 mM l-glutamine, 50 μM 2-mercaptoethanol, and 10 ng/mL murine GM-CSF. The medium was replenished on days 3 and 6, and the nonadherent cells were harvested on day 8.

### Plasmids.

To construct the dual GFP:nanoluciferase lentiviral vector plenti.GFP-NLuc, GFP was amplified from plenti.CMV.GFP.puro (Addgene 17448) with a forward primer containing a BamH-I site and a reverse primer encoding the picornavirus P2A amino acid motif, and the nanoluciferase coding sequence was amplified with a forward primer encoding P2A and a reverse primer containing a Sal-I site. All primers were provided by Integrated DNA Technologies. The fragments were joined by overlap extension PCR, digested with BamH-I and Sal-I, and ligated to BamH-I/Sal-I–digested plenti.CMV.GFP.puro (Addgene 17448) to remove the vector GFP gene and replace it with the GFP-P2A-nanoluciferase amplicon. To construct the murine CD40L-expressing lentiviral vector, plenti.CD40L, a murine CD40L cDNA, was amplified with primers containing flanking 5′-Xba-I and 3′-Sal-I sites, cleaved with Xba-I and Sal-I, and ligated to plenti.CMV.GFP.puro. To generate plenti.CD40L-GP33, mouse CD40L (mCD40L) was amplified with a forward primer containing an Xba-I site and reverse primer encoding the P2A motif and sequence encoding LCMV GP33-41 and a 3′-Sal-I site. The amplicon was cleaved with Xba-I and Sal-I and ligated to plenti.CMV.GFP.puro. To generate plenti.mut.mCD40L-GP33, an inactivating point mutation (T146N) was introduced into mCD40L by overlapping PCR. The mutated CD40L-GP33 was then amplified by PCR using the primers containing Xba-I and Sal-I sites and subcloned into plenti.CMV.GFP.puro. To construct plenti.CD40L-GP33.GP66, the adenovirus E6/gp19K signal peptide sequence MRYMILGLLALAAVCSAA was fused in-frame to sequence encoding the codon-optimized LCMV GP66-77 peptide DIYKGVYQFKSV flanked by Pst-I and Xho-I sites. The amplicon was cleaved with Pst-I and Xho-I and ligated to the PGK promoter of plenti.CD40L-GP33. To generate plenti.PD-1-CD40L, an amplicon was constructed expressing a codon-optimized PD-1 signal peptide fused to the PD-1 microbody sequence with a 5′-Xba-1 site, 3′-8(His)-tag, and P2A motif. To construct pCDH.mPD-L1, an amplicon was generated encoding P2A fused to mCD40L and containing a 3′-Sal-I site. An amplicon containing the PD-1 microbody fused to CD40L was generated by overlap extension PCR, cleaved with Xba-I and Sal-I, and ligated to plenti.CMV.GFP.puro.

### Lentiviral vector preparation.

Lentiviral vector stocks were prepared by calcium phosphate cotransfection of HEK293T cells with lentiviral vector plasmid, pMDL, pcVSV-G, and pcRev at a ratio of 28:20:7:5. Virus-containing supernatant was harvested 2 days after transfection and concentrated by ultracentrifugation for 90 minutes at 175,000*g* and 4°C in an SW32 rotor in a Beckman Coulter Optima L-100K ultracentrifuge. The virus was resuspended in 1/10 volume of PBS, frozen in aliquots, and titered on HEK293T cells by flow cytometry.

### Luciferase assay.

Mice (*n* = 3) were injected with 1 × 10^7^ IU GFP-NLuc virus via i.p. injection or i.v. injection. After 5 days, the mice were sacrificed and the tissues homogenized in cold PBS at 10% weight/volume in lysing matrix D tubes (MP Biomedicals) with a FastPrep-24 5G homogenizer (MP Biomedicals). The lysate was mixed with an equal volume of Nano-Glo Luciferase Assay Reagent (Promega), and luminescence was quantified on an EnVision 2103 Multi-label plate reader (PerkinElmer). For localization of transduced cells in live mice, infected mice (*n* = 3–6) were injected i.p. with 100 μL of 1:40 diluted Nano-Glo substrate and, after 3 minutes, imaged on an IVIS Lumina III XR (PerkinElmer).

### Antibodies and flow cytometry.

All antibody information is shown in Supplemental Table 1. Splenocytes were treated for 30 minutes at 4°C with anti-CD16/CD32 mAb to block Fc receptors and then stained with Fixable Viability Dye eFluor 450 (eBioscience) followed by labeled antibody. Antibodies used for cell surface staining were Alexa Fluor 700–anti-CD3 (BD Biosciences); PerCP Cy5.5 anti-CD8a, APC-Cy7 anti-CD4, APC CD11c, PerCP Cy5.5–anti-CD11b, PE-Cy7–anti-CD19, APC-Cy7–anti–I-A/I-E (MHC II), and BV421 or APC H-2b KAVYNFATC GP33 tetramer (NIH Tetramer Core); and PE–anti-CTLA-4 and PE/Cy7–anti-TIM3 (BioLegend). For intracellular staining, the cells were fixed for 10 minutes in 4% paraformaldehyde, then permeabilized with PBS/0.1% saponin prior to antibody addition. Antibodies used in intracellular staining were PE–anti–IFN-γ and APC-Cy7–anti–TNF-α. Cytokine levels were measured in 10 μL serum by cytokine bead array using the Mouse Inflammation Cytometric Bead Array Kit (BD Biosciences). The cells were analyzed by flow cytometry on a BD Biosciences LSR-II and the data analyzed with FlowJo software.

### Prophylactic and therapeutic vaccination.

For prophylactic vaccination, C57BL/6 (Taconic Biosciences) or SAMHD1-KO (provided by Axel Roers, Technische Universität Dresden, Dresden, Germany; ref. 52) mice were injected i.p. or i.v. with 2.5 × 10^6^ IU lentiviral vector. Seven days postvaccination, the mice were injected with 2.0 × 10^5^ PFU LCMV Armstrong (provided by Dirk Homann, Mount Sinai, New York, New York, USA). Four days postinfection, splenic virus load was measured by real-time qRT-PCR. For chronic LCMV infection, SAMHD1-KO mice were challenged by i.v. injection of 5 × 10^6^ PFU LCMV clone 13. Two weeks postinfection, the mice were injected with 3 × 10^6^ IU of lentivirus. Serum was collected weekly and virus load was measured. One week postinjection, the transduced BMDCs were injected i.p. or i.v. Where indicated, the mice were injected 3 times every third day with 30 to 50 μg anti–PD-L1 antibody (Bio X Cell), clone 10F.9G2, BP0101).

### Adoptive transfer.

C57BL/6 mice were immunized by i.v. injection of 3 × 10^6^ IU of lentiviral vector. One week postimmunization, the mice were sacrificed and the spleens were harvested. CD8^+^ T cells, B cells, and DCs were isolated on CD8, CD19, and CD11c MicroBeads UltraPure (Miltenyi Biotec), respectively. Mice were injected i.v. with 1 × 10^6^ each cell type. Five days postinjection, the mice were infected by i.p injection of 2 × 10^5^ PFU of LCMV Armstrong. After 4 days, the splenic virus load was measured by real-time qRT-PCR. To trace the fate of the transferred cells, splenocytes were labeled for 20 minutes at 37°C with 5 μM of CellTrace CFSE (Invitrogen). Labeling was quenched by a 5-minute incubation in RPMI 1640/10% FBS. A total of 1 × 10^4^ labeling cells were then injected. The mice were sacrificed 1 day later. Spleen, lymph node, thymus, and PBMCs were harvested, and the labeled cells were quantified by flow cytometry.

### Virus load quantification.

Mouse spleen and kidney were homogenized in FastPrep-24 5G homogenizer, and RNA was prepared from 200 μL lysate with a Quick-RNA MiniPrep kit (Zymo Research). The RNA (2 μg) was mixed with TaqMan Fast Virus 1-Step Master Mix (Applied Biosystems), forward and reverse primers (10 μM), and probe (2 μM) and amplified by qRT-PCR (50°C/5 min, 95°C/20 s and 40 cycles of 95°C/3 s, 60°C/30 s). Primers were LCMV.GP-forward (5′-GGCACATTCACCTGGACTTTG-3′) and LCMV.GP-reverse (5′-CTGCTGTGTTCCCGAAACACT-3′), and the probe was Fam/ZEN/IBFQ LCMV.GP (5′-ACTCTTCAGGGGTGGAGAATCCAGGTGGTT-3′). The data were normalized to GAPDH amplified with primers mu-GAPDH.forward (5′-CAATGTGTCCGTCGTGGATCT-3′) and mu-GAPDH.reverse (5′-GTCCTCAGTGTAGCCCAAGATG-3′) and mu-GAPDH probe (5′-CGTGCCGCCTGGAGAAACCTGCC-3′). The viral RNA copy number was determined using a standard curve generated with serially diluted pcDNA6-LCMV GP plasmid that contains a cloned copy of the LCMV target region. The data were normalized to GAPDH. To measure virus load in the serum, RNA was extracted from 50 μL sera and amplified as described above without the GAPDH step. Virus load was determined by the 2^−ΔΔCT^ method.

### In vitro cytolysis assay.

For LCMV cytolysis, LB27.4 target cells were stained with 5 μM of CellTrace CFSE for 20 minutes at 37°C and quenched for 5 minutes at 37°C in RPMI 1640/10% FBS. The cells (1 × 10^4^) were plated in a 96-well plate and coated for 2 hours with 1 ng/μL GP33 peptide. Splenocytes, CD8^+^ T cells, CD8^–^ T cells, DCs, and B cells (1, 3, 10, 20, 30, and 50 × 10^5^) were added. Effector cells were added after 6 hours of incubation. After 24 hours, the cells were stained with Fixable Viability Dye eFluor 450 (Thermo Fisher Scientific). Dead CFSE^+^ cells were quantified by flow cytometry on an LSR-II, and the data were analyzed with FlowJo software.

### Statistics.

All experiments were done 2 or 3 times with similar results. Statistical significance was determined by 2-tailed, unpaired *t* test. Statistical significance was calculated with GraphPad Prism 7 7.0e. Significance was based on 2-sided *t* testing and assigned at *P* < 0.05. Confidence intervals are shown as the mean ± SD.

### Study approval.

Animal research was performed under the written approval of the NYU Animal Research Committee in compliance with federal, state, and local guidelines.

## Author contributions

TT, TDN, and NRL designed the experiments. TT and TDN wrote the manuscript. TT, TDN, and RL carried out the experiments. NRL directed the study, planned the experiments, and edited the manuscript.

## Supplementary Material

Supplemental data

## Version 1 08/16/2022

In-Press Preview

## Version 2 09/22/2022

Electronic publication
