# Supplementary material for: Directly injected lentiviral vector–based T cell vaccine protects mice against acute and chronic viral infection
Source: JCI Insight. 2022 Sep 22;7(18):e161598. doi: 10.1172/jci.insight.161598 (PMC9675446; doi:10.1172/jci.insight.161598)
Supplement: Supplemental data [file jciinsight-7-161598-s153.pdf]

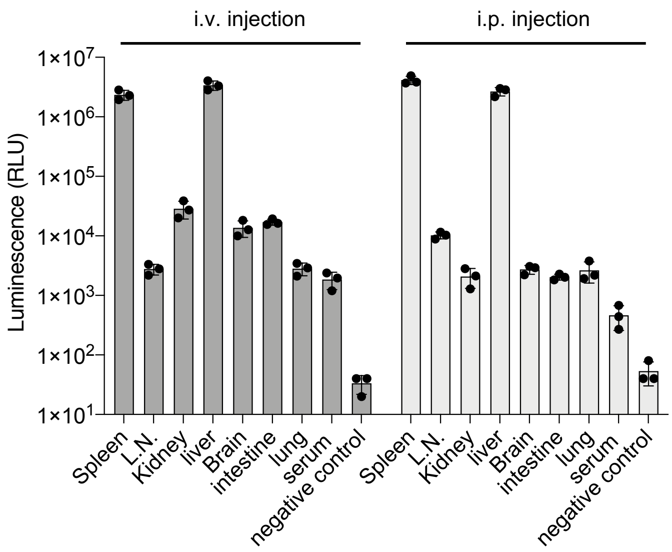

Supplementary Figure 1. **Direct lentiviral vector via IV injection migrated into spleen.**

(A) 1×10^7 IU of VSV-G-pseudotyped GFP-NLuc virions were directly injected into SAMHD1 KO mice (n=3) via intraperitoneal (i.p.) or intravenous (i.v.) injection. Three days post injection, mice were sacrificed and luminescence in the different organs was quantified by nano-Glo assay.

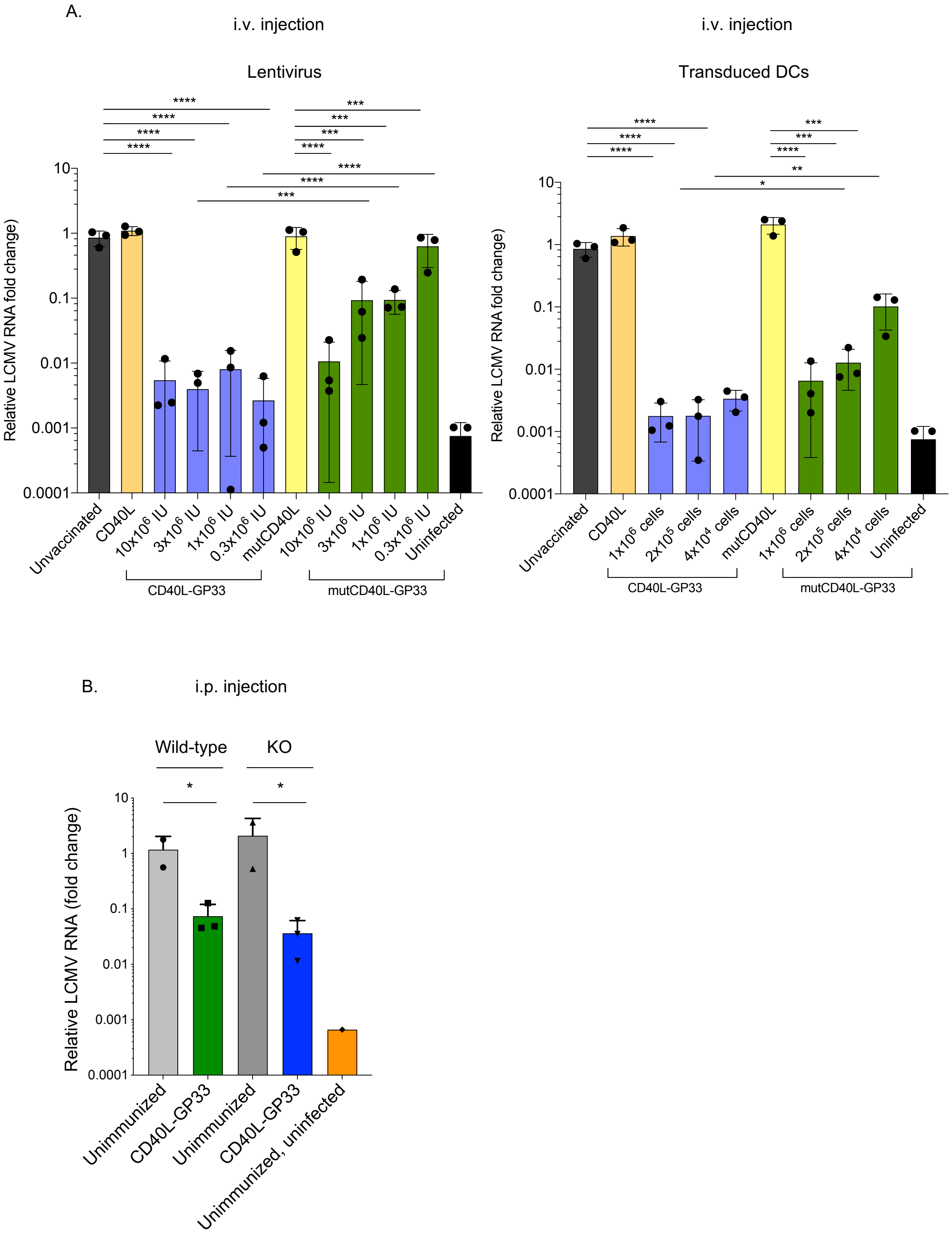

Supplementary Figure 2. A small amount of lentivirus prevented acute LCMV infection.

(A) SAMHD1 KO mice (n=4) were injected i.v. with 0.3-10 × 10^6 IU CD40L-GP33 or mutated CD40L-GP33 (mutCD40L-GP33) (left). In comparison to direct lentivirus injection, SAMHD1 knockout BMDCs were transduced with CD40L or CD40L-GP33 or mutCD40L-GP33 at MOI=5 and then different cell numbers were injected into mice (right). One week post-immunization, the mice were challenged with LCMV Armstrong. Four days post-infection, LCMV RNA copy numbers in the spleen were quantified by RT-PCR.

(B) Wild-type and SAMHD1 knockout mice (n=3 each) were injected with 3 × 10^6 IU of lentivirus. One week post-immunization, the mice were challenged with LCMV Armstrong. Four days post-infection, viral RNA copy numbers were determined by RT-PCR. Statistical significance was determined by two-tailed, unpaired t test. Confidence intervals are shown as the mean ± SD. (*P ≤ 0.05, **P ≤ 0.01, ***P≤0.001, ****P≤0.0001).

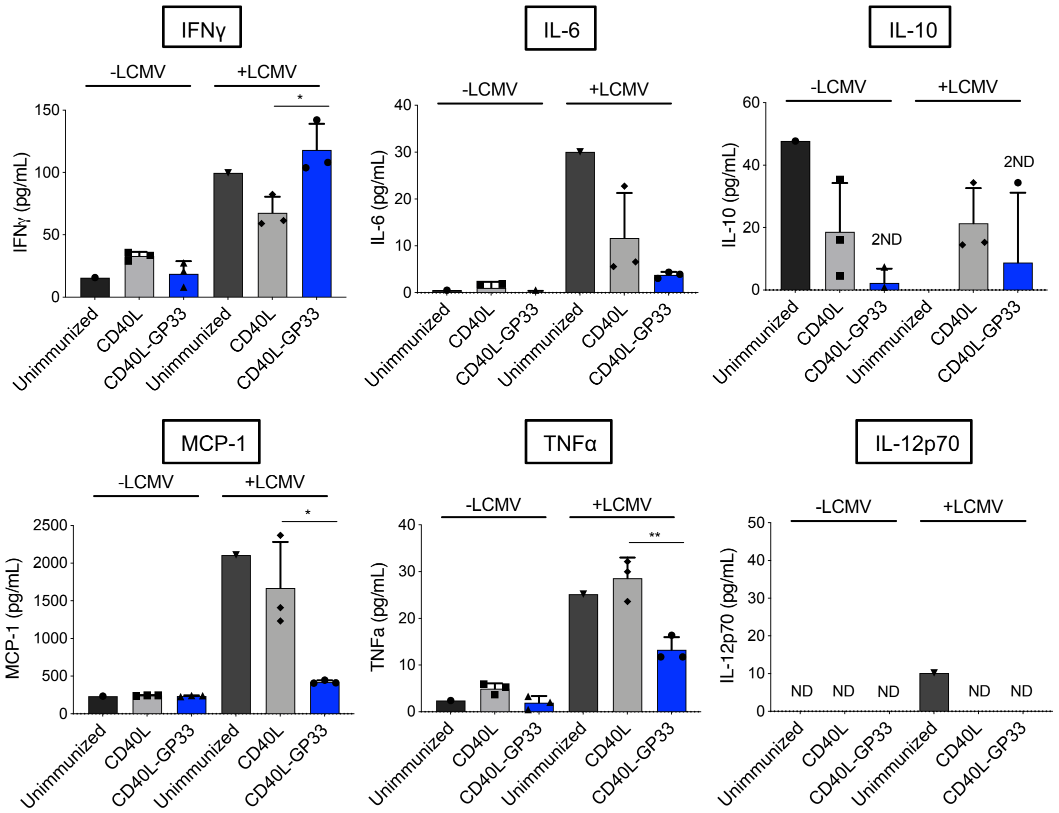

Supplementary Figure 3. T cells activated by vaccination with CD40L-GP33 induced cytokine secretion.

SAMHD1 KO mice (n=3) were immunized by i.v. injection of CD40L or CD40L-GP33 vectors. Mice were bled prior to immunization and 2 weeks post-immunization and serum proinflammatory cytokine levels of IFNγ, IL-6, IL-10, MCP-1, TNFα and IL-12p70 were measured by CBA. Statistical significance was determined by two-tailed, unpaired t test. Confidence intervals are shown as the mean ± SD. (*P ≤ 0.05, **P ≤ 0.01).

Supplemental Table 1. Antibody information used in this study.

Antibody	Source	Clone	Provider	Catalog number
anti-CD16/CD32	Mouse	Clone 93	BioLegend	101320
Viability dye eFluor 450	Mouse	-	eBiosciences	65-0863-14
Alexa 700 anti-CD3	Mouse	17A2	BD biosciences	100216
PerCP Cy5.5 anti-CD8a	Mouse	53-6.7	BioLegend	100734
APC-Cy7 anti-CD4	Mouse	GK1.5	BioLegend	100414
APC anti-CD11c	Mouse	N418	BioLegend	117309
PerCP Cy5.5 anti-CD11b	Mouse	M1/70	BioLegend	101227
PE-Cy7 anti-CD19	Mouse	6D5	BioLegend	115519
APC-Cy7 anti-I-A/I-E (MHC II)	Mouse	M5/114.15.2	BioLegend	107628
BV421 H-2b KAVYNFATC GP33 tetramer	Mouse	-	NIH tetramer core	-
APC H-2b KAVYNFATC GP33 tetramer	Mouse	-	NIH tetramer core	-
PE anti-IFNγ	Mouse	XMG1.2	BioLegend	505808
APC-Cy7 anti-TNFα	Mouse	MP6-XT22	BioLegend	506343
PE anti-CTLA-4	Mouse	UC10-4B9	BioLegend	106305
PE/Cy7 anti-TIM3	Mouse	B8.2C12	BioLegend	134009
